# Supplementary material for: A Hybrid Approach Combining Shape-Based and Docking Methods to Identify Novel Potential P2X7 Antagonists from Natural Product Databases
Source: Pharmaceuticals (Basel). 2024 May 7;17(5):592. doi: 10.3390/ph17050592 (PMC11123696; doi:10.3390/ph17050592)
Supplement: Supplementary file 1 [file pharmaceuticals-17-00592-s001.zip › Supplementary Material.pdf]

Sequence alignment between *Ailuropoda melanoleuca* P2X7 (*Am*P2X7) and *Homo sapiens* P2X7 (*Hs*P2X7) using BLAST

```
RID: P9XTZYJU114
Job Title:tr|G1M6C4|G1M6C4_AILME ATP receptor OS=Ailuropoda...
Program: BLASTP
Query: tr|G1M6C4|G1M6C4_AILME ATP receptor OS=Ailuropoda melanoleuca OX=9646
GN=P2RX7 PE=1 SV=2 ID: lcl|Query_50083(amino acid) Length: 606
Subject:sp|Q99572|P2RX7_HUMAN P2X purinoceptor 7 OS=Homo sapiens OX=9606 GN=P2RX7
PE=1 SV=4 ID: lcl|Query_50085(amino acid) Length: 595

Sequences producing significant alignments:
                                                                        Scientific
Common                         Max     Total Query   E    Per.   Acc.
Description                                                             Name
Name            Taxid        Score   Score cover Value Ident  Len         Accession
sp|Q99572|P2RX7_HUMAN P2X purinoceptor 7 OS=Homo sapiens OX=96...
0          1060    1060   98%    0.0    84.71  595          Query_50085
Alignments:
>sp|Q99572|P2RX7_HUMAN P2X purinoceptor 7 OS=Homo sapiens OX=9606 GN=P2RX7 PE=1
SV=4
Sequence ID: Query_50085 Length: 595
Range 1: 1 to 595
Score:1060 bits(2742), Expect:0.0,
Method:Compositional matrix adjust.,
Identities:504/595(85%), Positives:545/595(91%), Gaps:1/595(0%

Query  1    MPACCSWKDVFQYETNKVLRIQSTNYGTIKWIFHVLVFSYISFALISDKRYQKKEPLISS  60
            MPACCS  DVFQYETNKV RIQS NYGTIKW FHV++FSY+ FAL+SDK YQ+KEP+ISS
Sbjct  1    MPACCSCSDVFQYETNKVTRIQSMNYGTIKWFFHVIIFSYVCFALVSDKLYQRKEPVISS  60

Query  61   VHTKVKGIAEVKAEILENGMKKMVSGVFDTADYTFPLQGNSFFVMTNFIKTEGQQQGLCP  120
            VHTKVKGIAEVK EI+ENG+KK+V  VFDTADYTFPLQGNSFFVMTNF+KTEGQ+Q LCP
Sbjct  61   VHTKVKGIAEVKEEIVENGVKKLVHSVFDTADYTFPLQGNSFFVMTNFLKTEGQEQRLCP  120

Query  121  DFPTRRTICSSDRGCKKGRMDPQSKGIQTGRCVVYKERLKTCEVSAWCPIEEVEDAPRPA  180
            ++PTRRT+CSSDRGCKKG MDPQSKGIQTGRCVVY+   KTCEVSAWCPIE VE+APRPA
Sbjct  121  EYPTRRTLCSSDRGCKKGWMDPQSKGIQTGRCVVYEGNQKTCEVSAWCPIEAVEEAPRPA  180

Query  181  LLNSAENFTVLIKNNIDFPGHNYTTRNILPGVNITCTFHKTQNPQCPIFRLGDIFQETGD  240
            LLNSAENFTVLIKNNIDFPGHNYTTRNILPG+NITCTFHKTQNPQCPIFRLGDIF+ETGD
Sbjct  181  LLNSAENFTVLIKNNIDFPGHNYTTRNILPGLNITCTFHKTQNPQCPIFRLGDIFRETGD  240

Query  241  NFSDVAIQGGIMGIEIYWDCNLDGWFHHCRPKYSFRRLDDKTTNESLYPGYNFRYAKYYK  300
            NFSDVAIQGGIMGIEIYWDCNLD WFHHCRPKYSFRRLDDKTTN SLYPGYNFRYAKYYK
Sbjct  241  NFSDVAIQGGIMGIEIYWDCNLDRWFHHCRPKYSFRRLDDKTTNVSLYPGYNFRYAKYYK  300

Query  301  ENNVEKRTLIKVFGIRFDILVFGTGGKFNVIQLAVYIGSVISYFGLATVFIDILINTYSS  360
            ENNVEKRTLIKVFGIRFDILVFGTGGKF++IQL VYIGS +SYFGLA VFID LI+TYSS
Sbjct  301  ENNVEKRTLIKVFGIRFDILVFGTGGKFDIIQLVVYIGSTLSYFGLAAVFIDFLIDTYSS  360

Query  361  KCCRSRIYPCFKCCEYCAVNEYYYRKKSEPIAEPKPTLKYVSFVDETHIRMVDQQLLGKS  420
             CCRS IYP  KCC+ C VNEYYYRKK E I EPKPTLKYVSFVDE+HIRMV+QQLLG+S
Sbjct  361  NCCRSHIYPWCKCCQPCVVNEYYYRKKCESIVEPKPTLKYVSFVDESHIRMVNQQLLGRS  420

Query  421  LQNVKGEKVQRPSVDFTDLSRLSLSLCDPTPIPGQPEEMQLFSEEVTPRSSNSPDWCQCG  480
            LQ+VKG++V RP++DFTDLSRL L+L D  PIPGQPEE+QL  +E TPRS +SP WCQCG
Sbjct  421  LQDVKGQEVPRPAMDFTDLSRLPLALHDTPPIPGQPEEIQLLRKEATPRSRDSPVWCQCG  480

Query  481  HCLPSQLPESHRCLEELCCRKKAGACITTSEPFRKLVLSRQVLQFLLLYQEPLLVLDGNS  540
             CLPSQLPESHRCLEELCCRKK GACITTSE FRKLVLSR VLQFLLLYQEPLL LD +S
Sbjct  481  SCLPSQLPESHRCLEELCCRKKPGACITTSELFRKLVLSRHVLQFLLLYQEPLLALDVDS  540

Query  541  S-SRLRHCAYRCYTTWRFGSPDLADFAILPSCCRWRIRREFPKSEGQYTGFQSPY  595
            + SRLRHCAYRCY TWRFGS D+ADFAILPSCCRWRIR+EFPKSEGQY+GF+SPY
Sbjct  541  TNSRLRHCAYRCYATWRFGSQDMADFAILPSCCRWRIRKEFPKSEGQYSGFKSPY  595
```

Residues highlighted in yellow form the allosteric binding site that accommodates JNJ-47965567.
