# Supplementary material for: A Hybrid Approach Combining Shape-Based and Docking Methods to Identify Novel Potential P2X7 Antagonists from Natural Product Databases
Source: Pharmaceuticals (Basel). 2024 May 7;17(5):592. doi: 10.3390/ph17050592 (PMC11123696; doi:10.3390/ph17050592)
Supplement: Supplementary file 1 [file pharmaceuticals-17-00592-s001.zip › Table S1.pdf]

Table S1. Structures, inhibitory activities, and Shape Tanimoto score values of some known P2X7 receptor allosteric antagonists that were applied to the proposed shape-based model.

**Compound ID**(s)	**Structure**	**Inhibitory activity(ies) (IC_{50}, pIC_{50}, K_d, pA_2, or percentage of inhibition)**	**Reference(s)**	**Shape Tanimoto score value**
**8n**	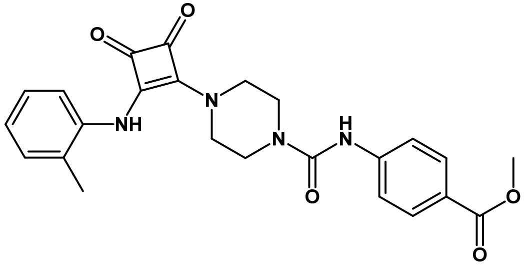	pIC_{50} = 6.99 ± 0.31	Patberg et al. *Eur. J. Med. Chem.*, 226, 113838, 2021.	0.694
**8o**	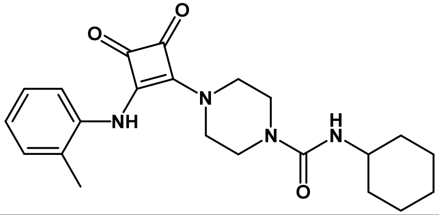	pIC_{50} = 6.13 ± 0.15	Patberg et al. *Eur. J. Med. Chem.*, 226, 113838, 2021.	0.693
**8m**	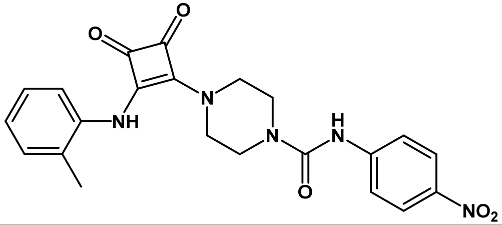	pIC_{50} = 7.13 ± 0.11	Patberg et al. *Eur. J. Med. Chem.*, 226, 113838, 2021.	0.693

**8l**	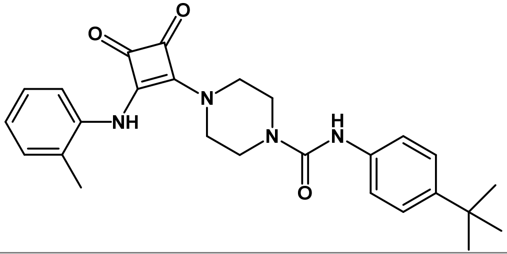	pIC_{50} = 6.96 ± 0.11	Patberg et al. *Eur. J. Med. Chem.*, 226, 113838, 2021.	0.691
**8j**	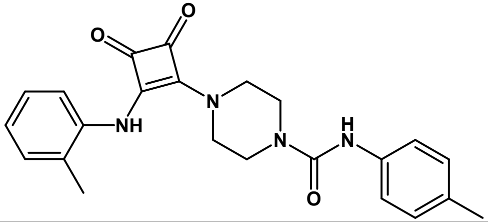	pIC_{50} = 6.51 ± 0.19	Patberg et al. *Eur. J. Med. Chem.*, 226, 113838, 2021.	0.668
**8i**	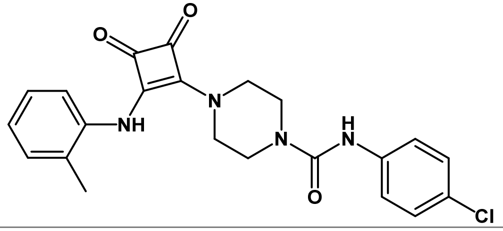	pIC_{50} = 6.80 ± 0.16	Patberg et al. *Eur. J. Med. Chem.*, 226, 113838, 2021.	0.668
**8h**	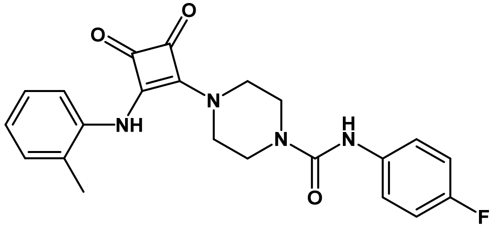	pIC_{50} = 6.12 ± 0.09	Patberg et al. *Eur. J. Med. Chem.*, 226, 113838, 2021.	0.668

**8g**	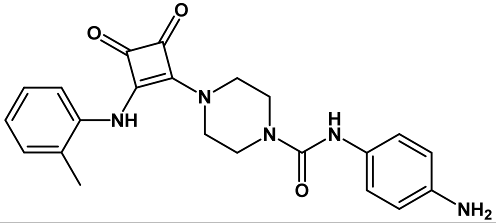	pIC_{50} = 6.42 ± 0.15	Patberg et al. *Eur. J. Med. Chem.*, 226, 113838, 2021.	0.668
**8p**	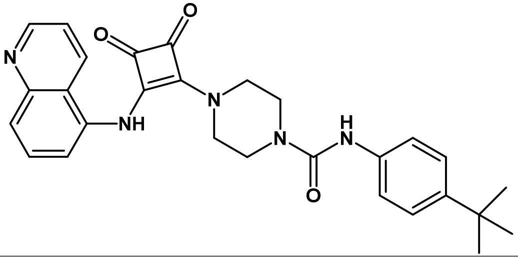	pIC_{50} = 7.80 ± 0.09	Patberg et al. *Eur. J. Med. Chem.*, 226, 113838, 2021.	0.667
**AZ11645373**	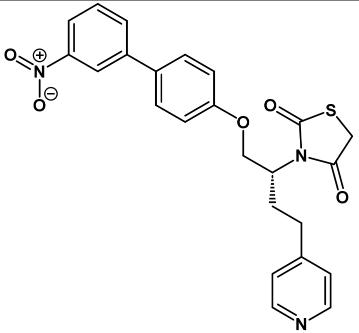	IC_{50} = 5 – 90 nM, depending on the type of assay.	Stoke et al. *Br. J. Pharmacol.*, 149, 880-887, 2006.<br>Michel et al. *Br. J. Pharmacol.*, 156, 1312-1325, 2009.<br>Dayel et al. *Mol. Pharmacol.*, 96, 355-363, 2019.	0.646
**8k**	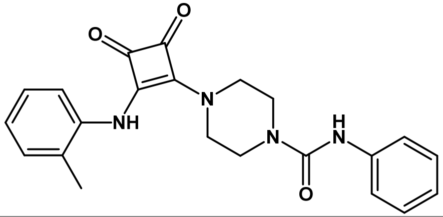	pIC_{50} = 6.82 ± 0.10	Patberg et al. *Eur. J. Med. Chem.*, 226, 113838, 2021.	0.645

**8f**	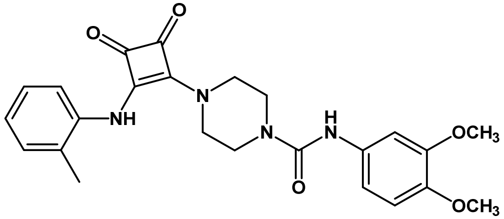	pIC_{50} = 6.52 ± 0.18	Patberg et al. *Eur. J. Med. Chem.*, 226, 113838, 2021.	0.643
**4n**	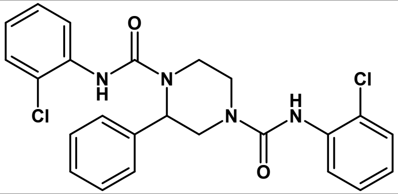	pIC_{50} = 6.31 ± 0.18	Patberg et al. *Eur. J. Med. Chem.*, 226, 113838, 2021.	0.638
**9o**	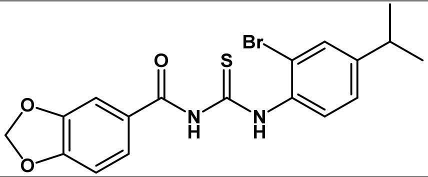	IC_{50} = (13.11 ± 0.25) µM	Mahmood et al. *Eur. J. Med. Chem.*, 238, 114491, 2022.	0.630
**Compound 4**	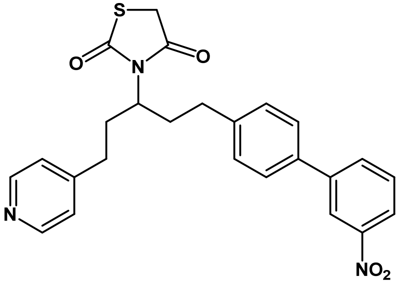	pA_2 = 6.9	Alcaraz et al. *Bioorg. Med. Chem. Lett.*, 13, 4043-4046, 2003. Jackson et al. *Eur. J. Med. Chem.*, 914, 174667, 2022.	0.626
**19**	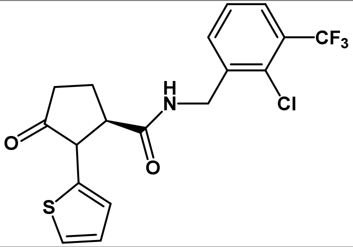	IC_{50} = (100.0 ± 10.1) nM	Homerin et al. *J. Med. Chem.*, 63, 2074-2094, 2020.	0.626

**Compound-17**<br>**AZ10606120**	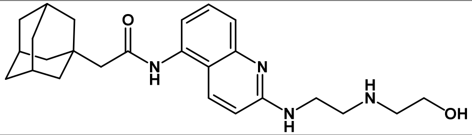	IC_{50} = 231 nM	Michel et al. Br. *J. Pharmaco*l., 153, 737-750, 2008.<br>Karasawa & Kawate. *eLife*, 5, e22153, 2016.	0.618
**9h**	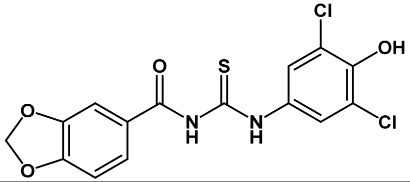	IC_{50} = (1.64 ± 0.07) μM	Mahmood et al. *Eur. J. Med. Chem.*, 238, 114491, 2022.	0.617
**A740003**	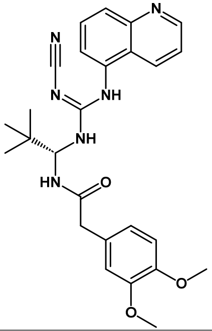	IC_{50} = 69.3 nM<br>pIC_{50} = 7.1 ± 0.05<br>(Against P2X7-2Nβ)	Karasawa & Kawate. *eLife*, 5, e22153, 2016.<br>Allsopp et al. *Mol. Pharmacol.*, 93, 553-562, 2018.	0.617
**9q**	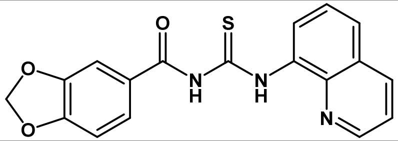	IC_{50} = (0.018 ± 0.06) μM	Mahmood et al. *Eur. J. Med. Chem.*, 238, 114491, 2022.	0.604
**GW791343**	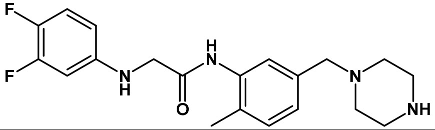	IC_{50} = 8.9 μM	Michel et al. Br. *J. Pharmacol.*, 153, 737-750, 2008.<br>Karasawa & Kawate. *eLife*, 5, e22153, 2016.	0.601

**16a**	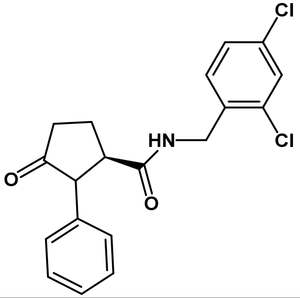	IC_{50} = (10000.0 ± 151.1) nM	Homerin et al. *J. Med. Chem.*, 63, 2074-2094, 2020.	0.598
**Calmidazolium**	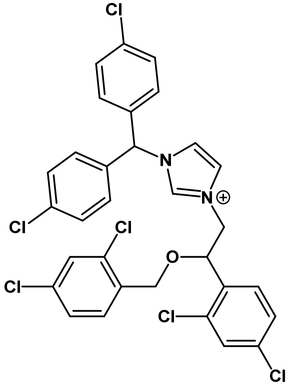	IC_{50} = 15 nM	Virgino et al. *Neuropharmacol.*, 36, 1285-1294, 1997.<br>Dayel et al. *Mol. Pharmacol.*, 96, 355-363, 2019.	0.597
**8d**	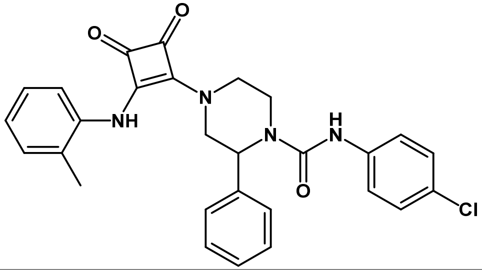	pIC_{50} = 6.87 ± 0.07	Patberg et al. *Eur. J. Med. Chem.*, 226, 113838, 2021.	0.595
**Z1456467176**	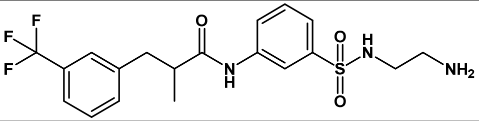	IC_{50} = 3.416 µM	Li et al. *Front. Pharmacol.*, 13, 979939, 2022.	0.594

**ZINC58368839**	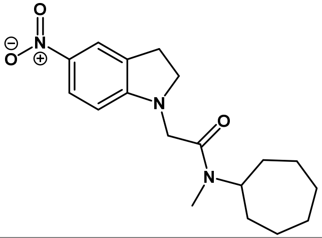	IC_{50} = 1.0 – 4.8 μM, depending on the type of assay.	Caseley et al. *Biochem. Pharmacol.*, 116, 130-139, 2016.<br>Dayel et al. *Mol. Pharmacol.*, 96, 355-363, 2019.	0.594
**8c**	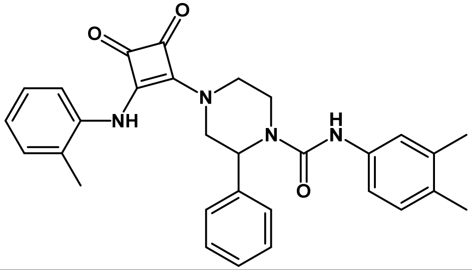	pIC_{50} = 7.19 ± 0.16	Patberg et al. *Eur. J. Med. Chem.*, 226, 113838, 2021.	0.594
**Compound 5**	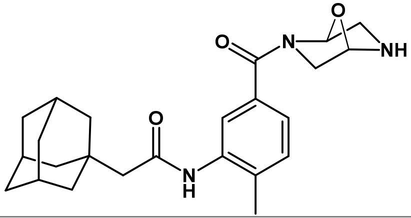	-	Jackson et al. *Eur. J. Med. Chem.*, 914, 174667, 2022.	0.588
**4i**	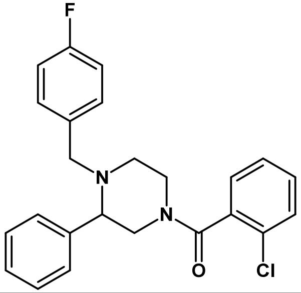	Inhibition (%, at 10 μM) = 35 ± 18	Patberg et al. *Eur. J. Med. Chem.*, 226, 113838, 2021.	0.583

**4m**	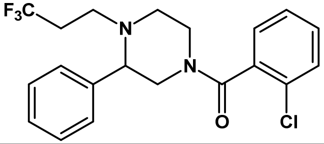	pIC_{50} = 6.00 ± 0.04	Patberg et al. *Eur. J. Med. Chem.*, 226, 113838, 2021.	0.582
**4d**	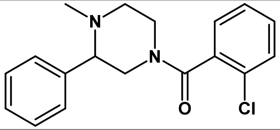	pIC_{50} = 4.31 ± 0.28	Patberg et al. *Eur. J. Med. Chem.*, 226, 113838, 2021.	0.577
**65**	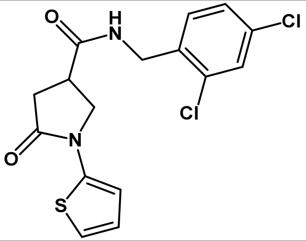	IC_{50} = (2406.0 ± 855.0) nM	Homerin et al. *J. Med. Chem.*, 63, 2074-2094, 2020.	0.571
**52a**	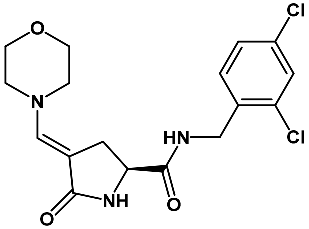	IC_{50} = (9557.0 ± 756.0) nM	Homerin et al. *J. Med. Chem.*, 63, 2074-2094, 2020.	0.567
**4o**	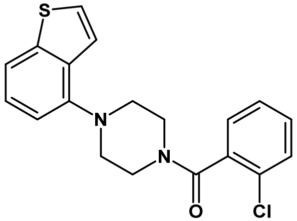	Inhibition (%, at 10 µM) = 34 ± 10	Patberg et al. *Eur. J. Med. Chem.*, 226, 113838, 2021.	0.567
**4l**	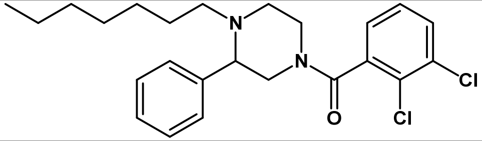	Inhibition (%, at 10 µM) = 19 ± 10	Patberg et al. *Eur. J. Med. Chem.*, 226, 113838, 2021.	0.567

**52b**	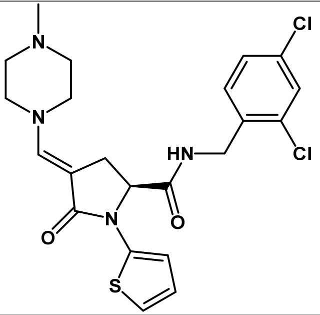	IC_{50} = (6947.0 ± 486.0) nM	Homerin et al. *J. Med. Chem.*, 63, 2074-2094, 2020.	0.566
**8e**	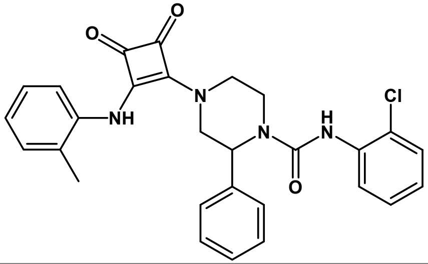	pIC_{50} = 7.06 ± 0.02	Patberg et al. *Eur. J. Med. Chem.*, 226, 113838, 2021.	0.566
**4f**	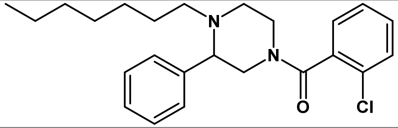	Inhibition (%, at 10 μM) = 4 ± 5	Patberg et al. *Eur. J. Med. Chem.*, 226, 113838, 2021.	0.566
**4c**	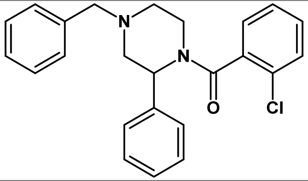	Inhibition (%, at 10 μM) = 14 ± 10	Patberg et al. *Eur. J. Med. Chem.*, 226, 113838, 2021.	0.564

**8a**	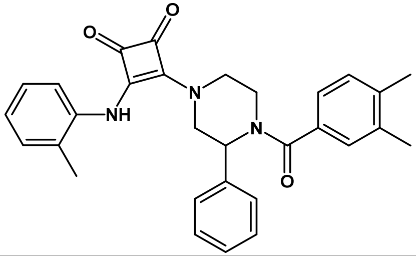	pIC_{50} = 6.26 ± 0.12	Patberg et al. *Eur. J. Med. Chem.*, 226, 113838, 2021.	0.563
**4e**	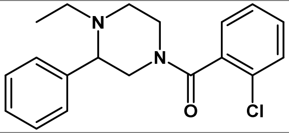	pIC_{50} = 4.08 ± 0.40	Patberg et al. *Eur. J. Med. Chem.*, 226, 113838, 2021.	0.563
**60**	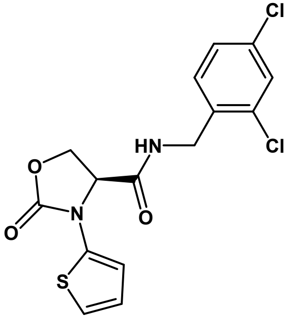	IC_{50} = (3168.0 ± 1023.0) nM	Homerin et al. *J. Med. Chem.*, 63, 2074-2094, 2020.	0.562
**4k**	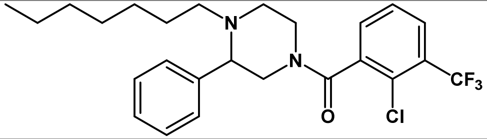	Inhibition (%, at 10 μM) = 29 ± 11	Patberg et al. *Eur. J. Med. Chem.*, 226, 113838, 2021.	0.558
**4h**	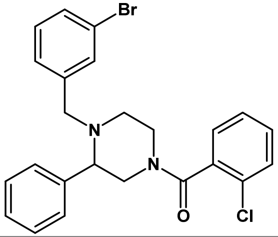	Inhibition (%, at 10 μM) = 29 ± 11	Patberg et al. *Eur. J. Med. Chem.*, 226, 113838, 2021.	0.558

**16n**	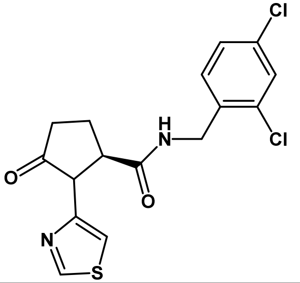	IC_{50} = (256.4 ± 1.0) nM	Homerin et al. *J. Med. Chem.*, 63, 2074-2094, 2020.	0.557
**4j**	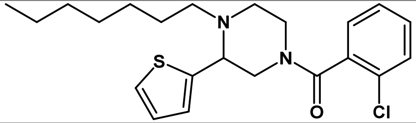	Inhibition (%, at 10 μM) = 31 ± 24	Patberg et al. *Eur. J. Med. Chem.*, 226, 113838, 2021.	0.557
**29c**	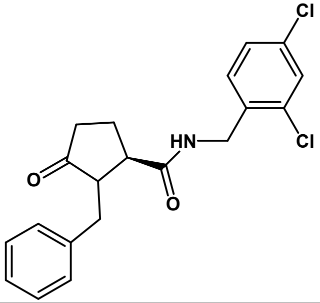	IC_{50} = (1130.3 ± 120.2) nM	Homerin et al. *J. Med. Chem.*, 63, 2074-2094, 2020.	0.556
**16l**	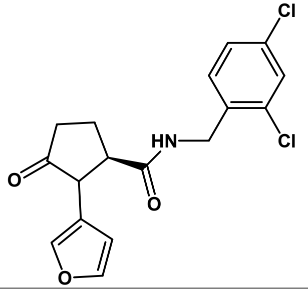	IC_{50} = (199.5 ± 15.5) nM	Homerin et al. *J. Med. Chem.*, 63, 2074-2094, 2020.	0.556

**16j**	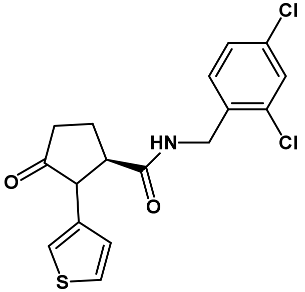	IC_{50} = (813.2 ± 56.7) nM	Homerin et al. *J. Med. Chem.*, 63, 2074-2094, 2020.	0.556
**16i**	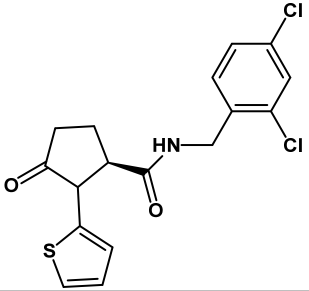	IC_{50} = (229.0 ± 4.5) nM	Homerin et al. *J. Med. Chem.*, 63, 2074-2094, 2020.	0.556
**8b**	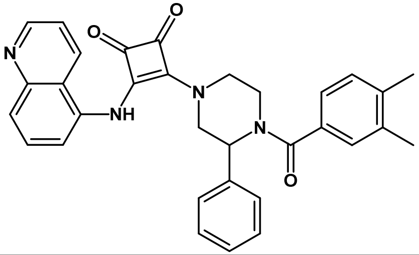	pIC_{50} = 6.65 ± 0.14	Patberg et al. *Eur. J. Med. Chem.*, 226, 113838, 2021.	0.556
**34**	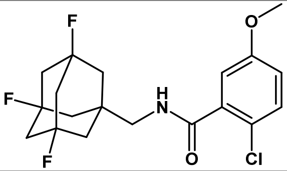	IC_{50} = (33.9 ± 11) nM	Wilkinson et al. *ACS Chem. Neurosci.*, 8, 2374-2380, 2017.	0.556

**16k**	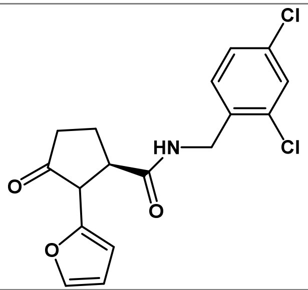	IC_{50} = (244.0 ± 105.0) nM	Homerin et al. *J. Med. Chem.*, 63, 2074-2094, 2020.	0.555
**39**	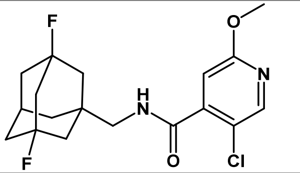	IC_{50} = (234 ± 30) nM	Wilkinson et al. *ACS Chem. Neurosci.*, 8, 2374-2380, 2017.	0.554
**16m**	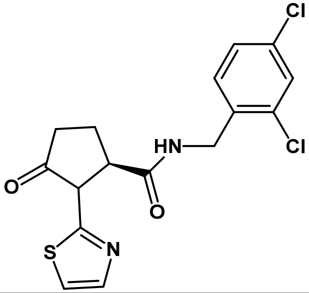	IC_{50} = (415.0 ± 85.0) nM	Homerin et al. *J. Med. Chem.*, 63, 2074-2094, 2020.	0.550
**71**	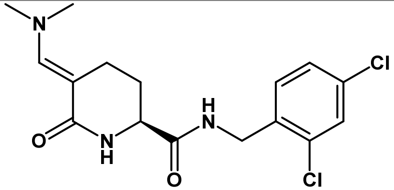	-	Homerin et al. *J. Med. Chem.*, 63, 2074-2094, 2020.	0.548

**4g**	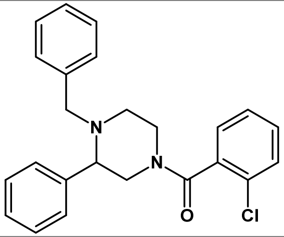	Inhibition (%, at 10 μM) = 21 ± 12	Patberg et al. *Eur. J. Med. Chem.*, 226, 113838, 2021.	0.547
**33**	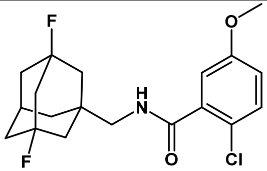	IC_{50} = (30.9 ± 8.0) nM	Wilkinson et al. *ACS Chem. Neurosci.*, 8, 2374-2380, 2017.	0.546
**51c**	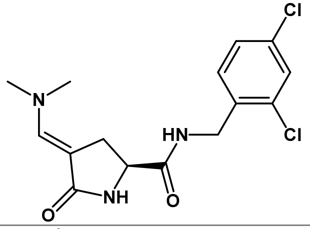	-	Homerin et al. *J. Med. Chem.*, 63, 2074-2094, 2020.	0.543
**Compound 1**	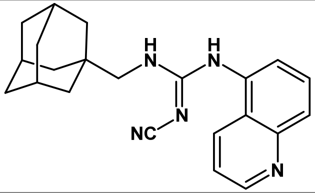	IC_{50} = (18 ± 2) nM	O’Brien-Brown et al. *Eur. J. Med. Chem.*, 130, 433-439, 2017. Jackson et al. *Eur. J. Med. Chem.*, 914, 174667, 2022.	0.539
**PS10**	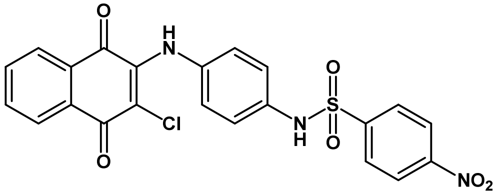	IC_{50} = (0.010 ± 0.002) μM	Pacheco et al. *Molecules*, 28, 590, 2023.	0.534

**50g**	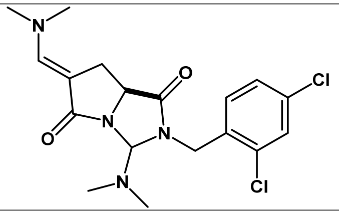	-	Homerin et al. *J. Med. Chem.*, 63, 2074-2094, 2020.	0.531
**28**	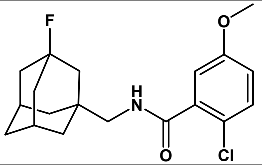	IC_{50} = (25.1 ± 2.7) nM	Wilkinson et al. *ACS Chem. Neurosci.*, 8, 2374-2380, 2017.	0.531
**1b**	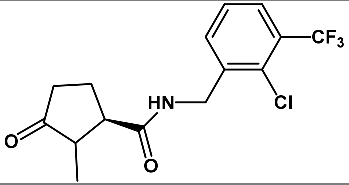	IC_{50} = (119.3 ± 7.0) nM	Homerin et al. *J. Med. Chem.*, 63, 2074-2094, 2020.	0.529
**4b**	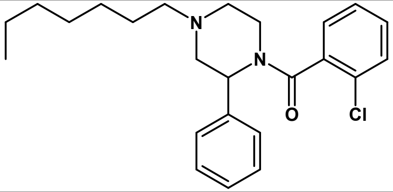	Inhibition (%, at 10 μM) = -11 ± 14	Patberg et al. *Eur. J. Med. Chem.*, 226, 113838, 2021.	0.528
**GSK1482160**	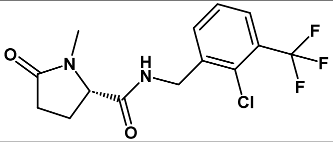	K_d = (1.15 ± 0.12) nM	Territo et al. *J. Nucl. Med.*, 58, 458-465, 2007.<br>Ali et al. Br. *J. Clin. Pharmacol.*, 75, 197-207, 2012.	0.528
**1**	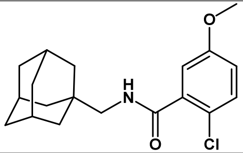	IC_{50} = (10.5 ± 3.1) nM<br>pA_2 = 8.8	Wilkinson et al. *ACS Chem. Neurosci.*, 8, 2374-2380, 2017.<br>Jackson et al. *Eur. J. Med. Chem.*, 914, 174667, 2022.	0.526

**9**	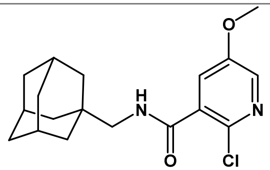	IC_{50} = (148 ± 25) nM	Wilkinson et al. *ACS Chem. Neurosci.*, 8, 2374-2380, 2017.	0.525
**2**	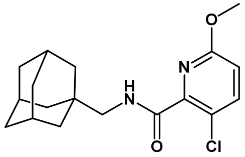	IC_{50} = (324 ± 78) nM	Wilkinson et al. *ACS Chem. Neurosci.*, 8, 2374-2380, 2017.	0.525
**13**	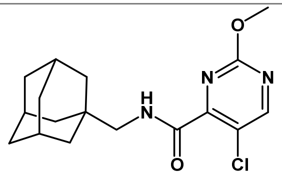	IC_{50} = (1410 ± 180) nM	Wilkinson et al. *ACS Chem. Neurosci.*, 8, 2374-2380, 2017.	0.524
**A804598**	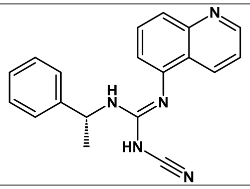	IC_{50} = 21.7 nM	Karasawa & Kawate. *eLife*, 5, e22153, 2016.	0.524
**17**	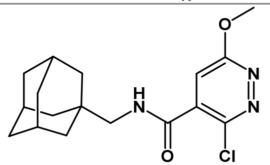	IC_{50} = (2240 ± 190) nM	Wilkinson et al. *ACS Chem. Neurosci.*, 8, 2374-2380, 2017.	0.523
**3**	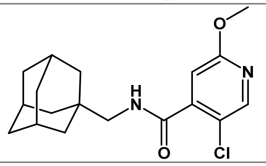	IC_{50} = (63.1 ± 9.4) nM	Wilkinson et al. *ACS Chem. Neurosci.*, 8, 2374-2380, 2017.	0.522

**1a**	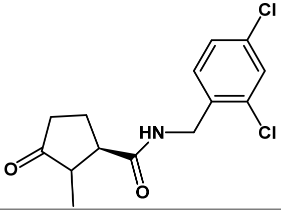	IC_{50} = (474.0 ± 12.0) nM	Homerin et al. *J. Med. Chem.*, 63, 2074-2094, 2020.	0.506
**30b**	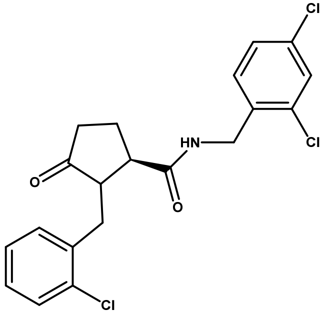	IC_{50} = (3461.2 ± 523.0) nM	Homerin et al. *J. Med. Chem.*, 63, 2074-2094, 2020.	0.501
**4a**	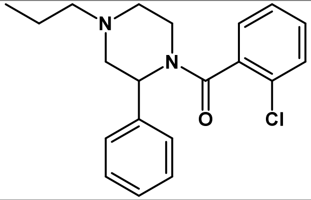	pIC_{50} = 4.08 ± 0.40	Patberg et al. *Eur. J. Med. Chem.*, 226, 113838, 2021.	0.485
**A438079**	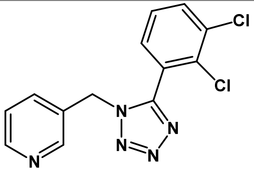	pIC_{50} = 6.0 ± 0.05 (Against P2X7-2Nβ)	Allsopp et al. *Mol. Pharmacol.*, 93, 553-562, 2018.	0.481

**Brilliant blue G**	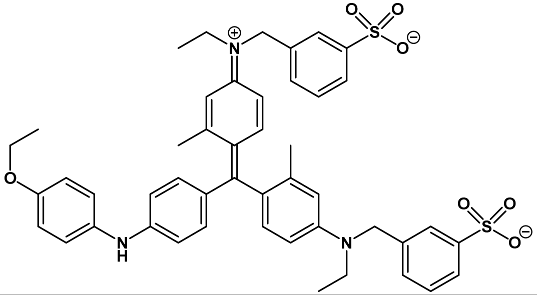	pIC$_{50}$ = 6.23 ± 0.10	Dayel et al. *Mol. Pharmacol.*, 96, 355-363, 2019.	0.470
**KN62**	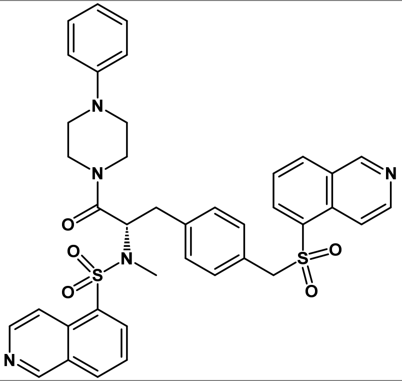	pIC$_{50}$ = 7.55 ± 0.09	Michel et al. *Br. J. Pharmacol.*, 151, 84-95, 2007.<br>Michel et al. *Br. J. Pharmacol.*, 153, 737-750, 2008.<br>Dayel et al. *Mol. Pharmacol.*, 96, 355-363, 2019.	0.467

<u>References</u>

Alcaraz, L.; Baxter, A.; Bent, J.; Bowers, K.; Braddock, M.; Cladingboel, D.; Donald, D.; Fagura, M.; Furber, M.; Laurent, C.; et al. Novel P2X7 Receptor Antagonists. *Bioorg Med Chem Lett* **2003**, *13*, 4043–4046, doi:10.1016/j.bmcl.2003.08.033.

Ali, Z.; Laurijssens, B.; Ostenfeld, T.; Mchugh, S.; Stylianou, A.; Scott-Stevens, P.; Hosking, L.; Dewit, O.; Richardson, J.C.; Chen, C. Pharmacokinetic and Pharmacodynamic Profiling of a P2X7 Receptor Allosteric Modulator GSK1482160 in Healthy Human Subjects. *Br J Clin Pharmacol* **2013**, *75*, 197–207, doi:10.1111/j.1365-2125.2012.04320.x.

Allsopp, R.C.; Dayl, S.; Dayel, A. Bin; Schmid, R.; Evans, R.J. Mapping the Allosteric Action of Antagonists A740003 and A438079 Reveals a Role for the Left Flipper in Ligand Sensitivity at P2X7 Receptorss. *Mol Pharmacol* **2018**, *93*, 553–562, doi:10.1124/mol.117.111021.

Caseley, E.A.; Muench, S.P.; Fishwick, C.W.; Jiang, L.H. Structure-Based Identification and Characterisation of Structurally Novel Human P2X7 Receptor Antagonists. *Biochem Pharmacol* **2016**, *116*, 130–139, doi:10.1016/j.bcp.2016.07.020.

Dayel, A. Bin; Evans, R.J.; Schmid, R. Mapping the Site of Action of Human P2X7 Receptor Antagonists AZ11645373, Brilliant Blue G, KN-62, Calmidazolium, and Zinc58368839 to the Intersubunit Allosteric Pocket. *Mol Pharmacol* **2019**, *96*, 355–363, doi:10.1124/mol.119.116715.

Homerin, G.; Jawhara, S.; Dezitter, X.; Baudelet, D.; Dufrénoy, P.; Rigo, B.; Millet, R.; Furman, C.; Ragé, G.; Lipka, E.; et al. Pyroglutamide-Based P2X7 Receptor Antagonists Targeting Inflammatory Bowel Disease. *J Med Chem* **2020**, *63*, 2074–2094, doi:10.1021/acs.jmedchem.9b00584.

Jackson, A.; Werry, E.L.; O'Brien-Brown, J.; Schiavini, P.; Wilkinson, S.; Wong, E.C.N.; McKenzie, A.D.J.; Maximova, A.; Kassiou, M. Pharmacological Characterization of a Structural Hybrid P2X7R Antagonist Using ATP and LL-37. *Eur J Pharmacol* **2022**, *914*, doi:10.1016/j.ejphar.2021.174667.

Janssen, B.; Vugts, D.J.; Wilkinson, S.M.; Ory, D.; Chalon, S.; Hoozemans, J.J.M.; Schuit, R.C.; Beaino, W.; Kooijman, E.J.M.; Van Den Hoek, J.; et al. Identification of the Allosteric P2X7 Receptor Antagonist [11C]SMW139 as a PET Tracer of Microglial Activation. *Sci Rep* **2018**, *8*, doi:10.1038/s41598-018-24814-0.

Karasawa, A.; Kawate, T. Structural Basis for Subtype-Specific Inhibition of the P2X7 Receptor. *eLife* **2016**, doi:10.7554/eLife.22153.001.

Li, X.; Liu, Y.; Luo, C.; Tao, J. Z1456467176 Alleviates Gouty Arthritis by Allosterically Modulating P2X7R to Inhibit NLRP3 Inflammasome Activation. *Front Pharmacol* **2022**, *13*, doi:10.3389/fphar.2022.979939.

Mahmood, A.; Villinger, A.; Iqbal, J. Therapeutic Potentials and Structure-Activity Relationship of 1,3-Benzodioxole N-Carbamothioyl Carboxamide Derivatives as Selective and Potent Antagonists of P2X4 and P2X7 Receptors. *Eur J Med Chem* **2022**, *238*, doi:10.1016/j.ejmech.2022.114491.

Michel, A.D.; Chambers, L.J.; Clay, W.C.; Condreay, J.P.; Walter, D.S.; Chessell, I.P. Direct Labelling of the Human P2X 7 Receptor and Identification of Positive and Negative Cooperativity of Binding. *Br J Pharmacol* **2007**, *151*, 84–95, doi:10.1038/sj.bjp.0707196.

Michel, A.D.; Chambers, L.J.; Walter, D.S. Negative and Positive Allosteric Modulators of the P2X 7 Receptor. *Br J Pharmacol* **2008**, *153*, 737–750, doi:10.1038/sj.bjp.0707625.

Michel, A.D.; Ng, S.W.; Roman, S.; Clay, W.C.; Dean, D.K.; Walter, D.S. Mechanism of Action of Species-Selective P2X 7 Receptor Antagonists. *Br J Pharmacol* **2009**, *156*, 1312–1325, doi:10.1111/j.1476-5381.2009.00135.x.

O'Brien-Brown, J.; Jackson, A.; Reekie, T.A.; Barron, M.L.; Werry, E.L.; Schiavini, P.; McDonnell, M.; Munoz, L.; Wilkinson, S.; Noll, B.; et al. Discovery and Pharmacological Evaluation of a Novel Series of Adamantyl Cyanoguanidines as P2X7 Receptor Antagonists. *Eur J Med Chem* **2017**, *130*, 433–439, doi:10.1016/j.ejmech.2017.02.060.

Pacheco, P.A.F.; Gonzaga, D.T.G.; von Ranke, N.L.; Rodrigues, C.R.; da Rocha, D.R.; da Silva, F. de C.; Ferreira, V.F.; Faria, R.X. Synthesis, Biological Evaluation and Molecular Modeling Studies of Naphthoquinone Sulfonamides and Sulfonate Ester Derivatives as P2X7 Inhibitors. *Molecules* **2023**, *28*, doi:10.3390/molecules28020590.

Patberg, M.; Isaak, A.; Füsser, F.; Ortiz Zacarías, N. V.; Vinnenberg, L.; Schulte, J.; Michetti, L.; Grey, L.; van der Horst, C.; Hundehege, P.; et al. Piperazine Squaric Acid Diamides, a Novel Class of Allosteric P2X7 Receptor Antagonists. *Eur J Med Chem* **2021**, *226*, doi:10.1016/j.ejmech.2021.113838.

Stokes, L.; Jiang, L.H.; Alcaraz, L.; Bent, J.; Bowers, K.; Fagura, M.; Furber, M.; Mortimore, M.; Lawson, M.; Theaker, J.; et al. Characterization of a Selective and Potent Antagonist of Human P2X 7 Receptors, AZ11645373. *Br J Pharmacol* **2006**, *149*, 880–887, doi:10.1038/sj.bjp.0706933.

Territo, P.R.; Meyer, J.A.; Peters, J.S.; Riley, A.A.; McCarthy, B.P.; Gao, M.; Min, W.; Green, M.A.; Zheng, Q.H.; Hutchins, G.D. Characterization of 11C-GSK1482160 for Targeting the P2X7 Receptor as a Biomarker for Neuroinflammation. *Journal of Nuclear Medicine* **2017**, *58*, 458–465, doi:10.2967/jnumed.116.181354.

Virginio, C.; Church, D.; North, R.A.; Surprenant, A. Effects of Divalent Cations, Protons and Calmidazolium at the Rat P2X7 Receptor. *Neuropharmacol* **1997**, 36 1285-1294, doi: 10.1016/s0028-3908(97)00141-x.

Wilkinson, S.M.; Barron, M.L.; O'Brien-Brown, J.; Janssen, B.; Stokes, L.; Werry, E.L.; Chishty, M.; Skarratt, K.K.; Ong, J.A.; Hibbs, D.E.; et al. Pharmacological Evaluation of Novel Bioisosteres of an Adamantanyl Benzamide P2X7 Receptor Antagonist. *ACS Chem Neurosci* **2017**, *8*, 2374–2380, doi:10.1021/acschemneuro.7b00272.
